# Predicting hospital mortality: comparing accuracy of SAPS II and clinical staff prognosis

**DOI:** 10.1186/cc11014

**Published:** 2012-03-20

**Authors:** I Patrício, M Marques, A Costa-Pereira, O Ribeiro, I Aragão, T Cardoso

**Affiliations:** 1 Hospital Geral de Santo António, University of Porto, Portugal; 2 Faculty of Medicine, University of Porto, Portugal

## Introduction

The purpose of this study is to compare the accuracy of Simplified Acute Physiology Score (SAPS) II with the subjective opinion of clinical staff in predicting hospital mortality, in critically ill adult patients.

## Methods

A prospective study in a mixed ICU, at a university hospital, using SAPS II to assess the risk of death. Patient outcome was also predicted subjectively by the clinical staff (consultants, residents and nurses), including the possibility of return to prior physical activity. The subjective predictions were compared with SAPS II predictions using logistic regression analysis and receiver operating characteristic curve (ROC) measurement, as well as sensitivity and specificity analysis for each group of participants.

## Results

**Figure 1:**
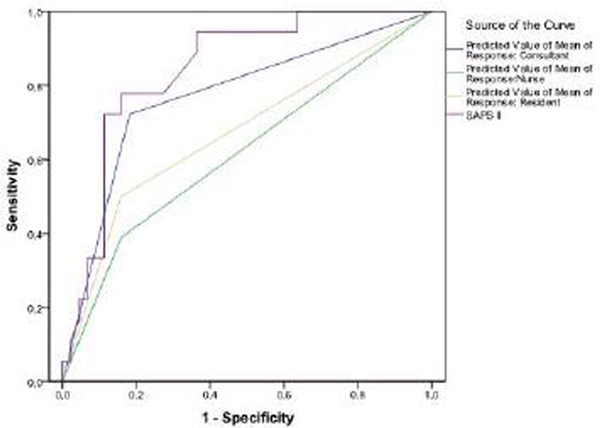
**ROC curve for SAPS II, consultants, nurses and residents, for hospital mortality**.

Over the study period 72 patients were included, with a mean age of 56.5 ± 16.8 years; 55% were male. The mean SAPS II was 47.3 ± 15.4. Eighteen patients died in hospital (25%). Discriminations analysis showed the following areas under ROC: SAPS II 0.84 (95% CI: 0.741 to 0.945); consultants 0.77 (95% CI: 0.632 to 0.908); residents 0.67 (95% CI: 0.513 to 0.828); nurses 0.62 (95% CI: 0.453 to 0.777). See Figure 1.

## Conclusion

In our study, contrary to previous descriptions of similar studies, SAPS II was more accurate in predicting hospital mortality than clinical staff opinion. Differences were also found between different groups of clinical staff, partially related to previous ICU clinical experience.
